# Supplementary material for: Genomic Analysis of Amphioxus Reveals a Wide Range of Fragments Homologous to Viral Sequences
Source: Viruses. 2023 Mar 31;15(4):909. doi: 10.3390/v15040909 (PMC10145014; doi:10.3390/v15040909)
Supplement: Supplementary file 1 [file viruses-15-00909-s001.zip › 909.pdf]

Supplementary Material for Genomic Analysis of Amphioxus Reveals a Wide Range of Fragments Homologous to Viral sequences

Authors: Qiao Du [1,†], Fang Peng [1,†], Qing Xiong [2,3,†], Kejin Xu [1], Kevin Yi Yang [2,3], Mingqiang Wang [2,4], Zhitian Wu [1], Shanying Li [1], Xiaorui Cheng [1], Xinjie Rao [1], Yuyouye Wang [1], Stephen Kwok-Wing Tsui [2,3] and Xi Zeng [1,*]

Affiliations:

[1] Agricultural Bioinformatics Key Laboratory of Hubei Province and 3D Genomics Research Centre, College of Informatics, Huazhong Agricultural University, Wuhan 430070, China
[2] School of Biomedical Sciences, The Chinese University of Hong Kong, Hong Kong, China
[3] Hong Kong Bioinformatics Centre, The Chinese University of Hong Kong, Hong Kong, China
[4] Stanford Cardiovascular Institute, Stanford University School of Medicine, Stanford, CA 94305, USA
* Correspondence: zengxi@mail.hzau.edu.cn; Tel.: +86-15818755917
† These authors contributed equally to this work.

This file includes:

Table S1 to S4

Figure S1 to S4

Table S1. The 17 viruses with viral HFs

Virus accession	Virus name	Number of HFs in viruses	Number of HFs in amphioxus	Number of HFs in viral CDS
NC_019491.1	*Cyprinid herpesvirus 1*	10	2	0
NC_021858.1	*Pandoravirus dulcis*	7	78	7
NC_026440.1	*Pandoravirus inopinatum*	7	73	3
NC_008168.1	*Choristoneura occidentalis granulovirus*	5	30	1
NC_006639.1	*Cotesia congregata bracovirus*	4	42	4
NC_026421.1	*Equid gammaherpesvirus 5*	3	10	0
NC_028045.1	*Tadarida brasiliensis circovirus 1*	2	37	0
NC_028094.1	*Chrysochromulina ericina virus*	2	8	0
NC_001716.2	*Human betaherpesvirus 7*	2	1	0
NC_001550.1	*Mason-Pfizer monkey virus*	1	29	0
NC_022098.1	*Pandoravirus salinus*	1	21	1
NC_026141.2	*Adelie penguin polyomavirus*	1	5	1
NC_008603.1	*Paramecium bursaria Chlorella virus* FR483	1	4	0
NC_008724.1	*Acanthocystis turfacea Chlorella virus 1*	1	4	1
NC_000852.5	*Paramecium bursaria Chlorella virus 1*	1	1	1
NC_008094.1	*Y73 sarcoma virus*	1	1	1
NC_023006.1	*Pseudomonas phage PPpW-3*	1	1	1

Table S2. The HFs in CDS regions of viral genomes

Virus accession	Virus name	Number of HFs in CDS	CDS annotation
NC_021858.1	*Pandoravirus dulcis*	7	Histone H2B domain containing protein
NC_006639.1	*Cotesia congregate bracovirus*	4	Histone (histone H4 like)
NC_026440.1	*Pandoravirus inopinatum*	3	hypothetical protein
NC_008094.1	*Y73 sarcoma virus*	1	protein-tyrosine kinase
NC_008168.1	*Choristoneura occidentalis granulovirus*	1	similar to Cydia pomonella granulovirus orf35 (hypothetical protein)
NC_008724.1	*Acanthocystis turfacea chlorella virus 1*	1	hypothetical protein
NC_000852.5	*Paramecium bursaria Chlorella virus 1*	1	hypothetical protein
NC_022098.1	*Pandoravirus salinus*	1	Histone H2B domain
NC_023006.1	*Pseudomonas phage PPpW-3*	1	PPpW-3_ORF-54 (hypothetical protein)
NC_026141.2	*Adelie penguin polyomavirus*	1	large T antigen

Table S3. The 10 amphioxus genes with the most HFs

Gene name (description)	Number of HFs	Product of gene
Histone H2B 1/2	78	Histone H2B 1/2
Histone H4	50	Histone H4
Late histone H2B.2.1	30	Histone H2B
Transposon TX1 uncharacterized 149 kDa protein	14	Transposon TX1 uncharacterized 149 kDa protein
Histone H2B (Fragments)	8	Histone H2B fragments
DCST1 (E3 ubiquitin-protein ligase DCST1)	7	E3 ubiquitin-protein ligase DCST1
H2BC13 (Histone H2B type 1-L)	7	Histone H2B type 1-L
DCST2 (DC-STAMP domain-containing protein 2)	6	DC-STAMP domain-containing protein 2
hist2h2l (Histone H2B 3)	6	Histone H2B 3
Histone H2B	4	Histone H2B

Table S4. The amphioxus genes with HFs in CDS regions

Gene name	Number of HFs in CDS	Number of HFs in gene
Histone H2B 1/2	77	78
Histone H4	45	51
Late histone H2B.2.1	30	30
H2BC13 (Histone H2B type 1-L)	7	7
Histone H2B (Fragments)	7	8
hist2h2l (Histone H2B 3)	6	6
Histone H2B	4	4
AGAP012199 (Histone H2B)	2	2
DCST1 (E3 ubiquitin-protein ligase DCST1)	2	7
H2AJ (Histone H2A.J)	2	2
H2bc3 (Histone H2B type 1-B)	2	2
Histone H3	2	2
H4DEKL (Histone H4)	1	1
Maob (Amine oxidase [flavin-containing] B)	1	1
Src42A (Tyrosine-protein kinase Src42A)	1	1
vit-6 (Vitellogenin-6)	1	1
VPS52 (Vacuolar protein sorting-associated protein 52 homolog)	1	1

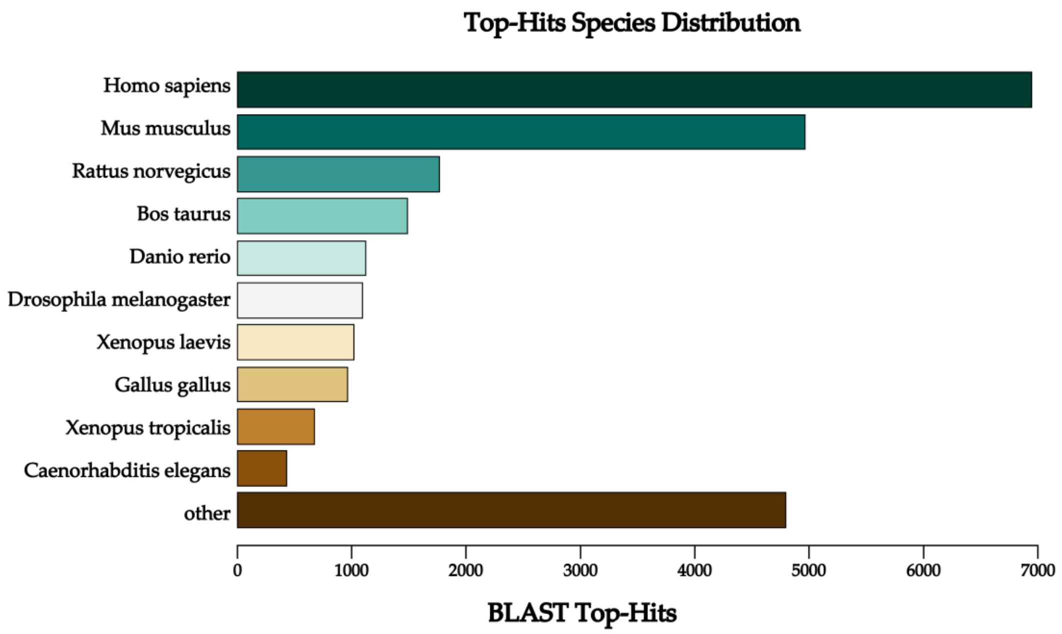

Figure S1. The species of top BLAST hits in functional annotation of the amphioxus genome.

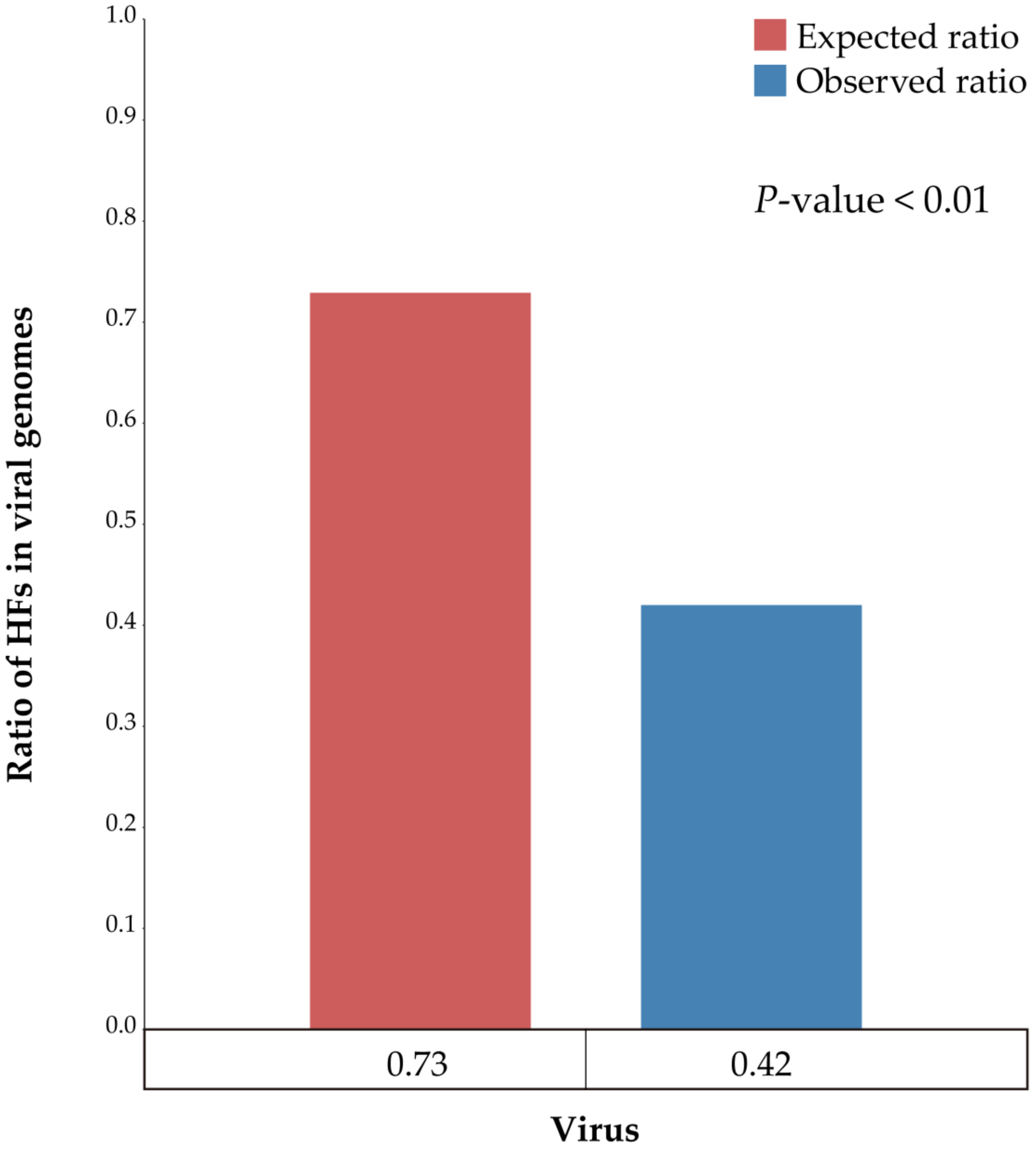

Figure S2. The distribution of HFs in CDS regions of viral genomes. The expected (random distribution, red) and the observed (actual numbers, blue) ratios of HFs in CDS regions are shown. The p value was calculated by X^2 test.

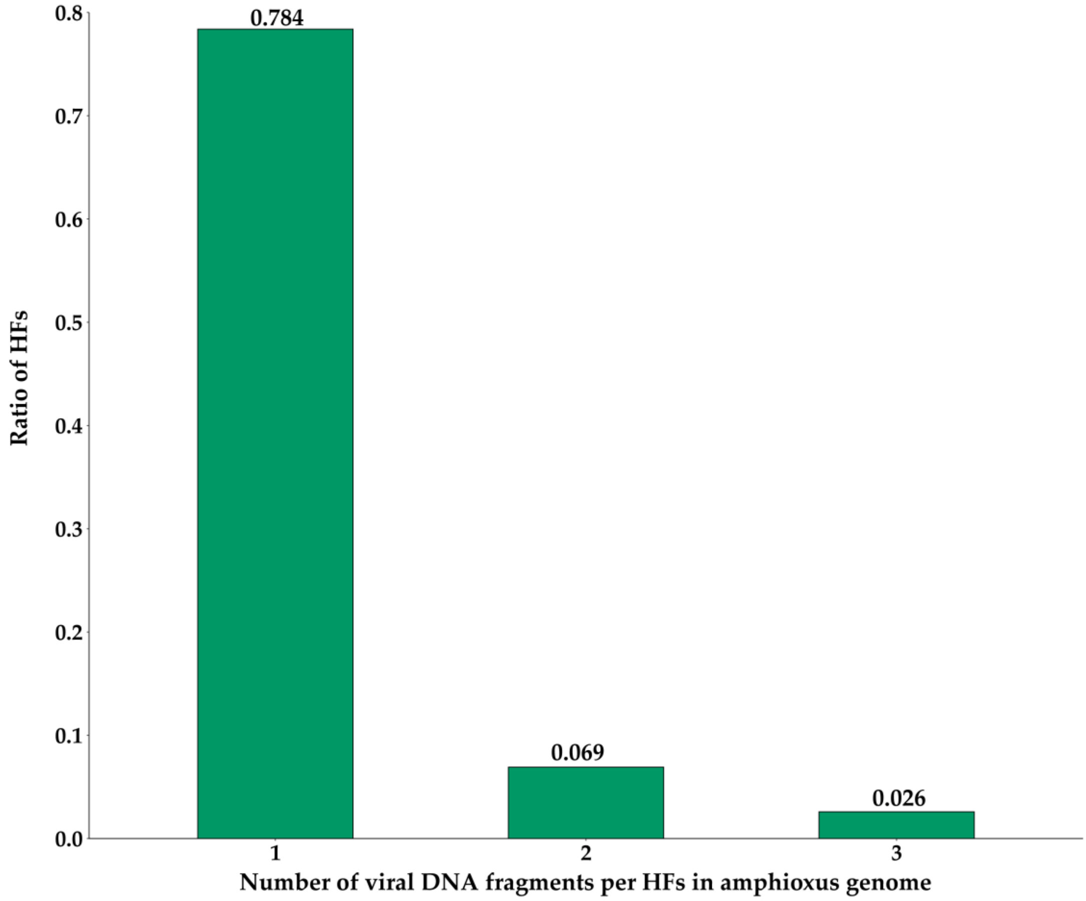

Figure S3. The number of viral DNA fragments which were homologous to each HF in the *B. belcheri* beihai genome. One HFs of the *B. belcheri* beihai genome could be homologous to more than one DNA fragment of viral genomes.

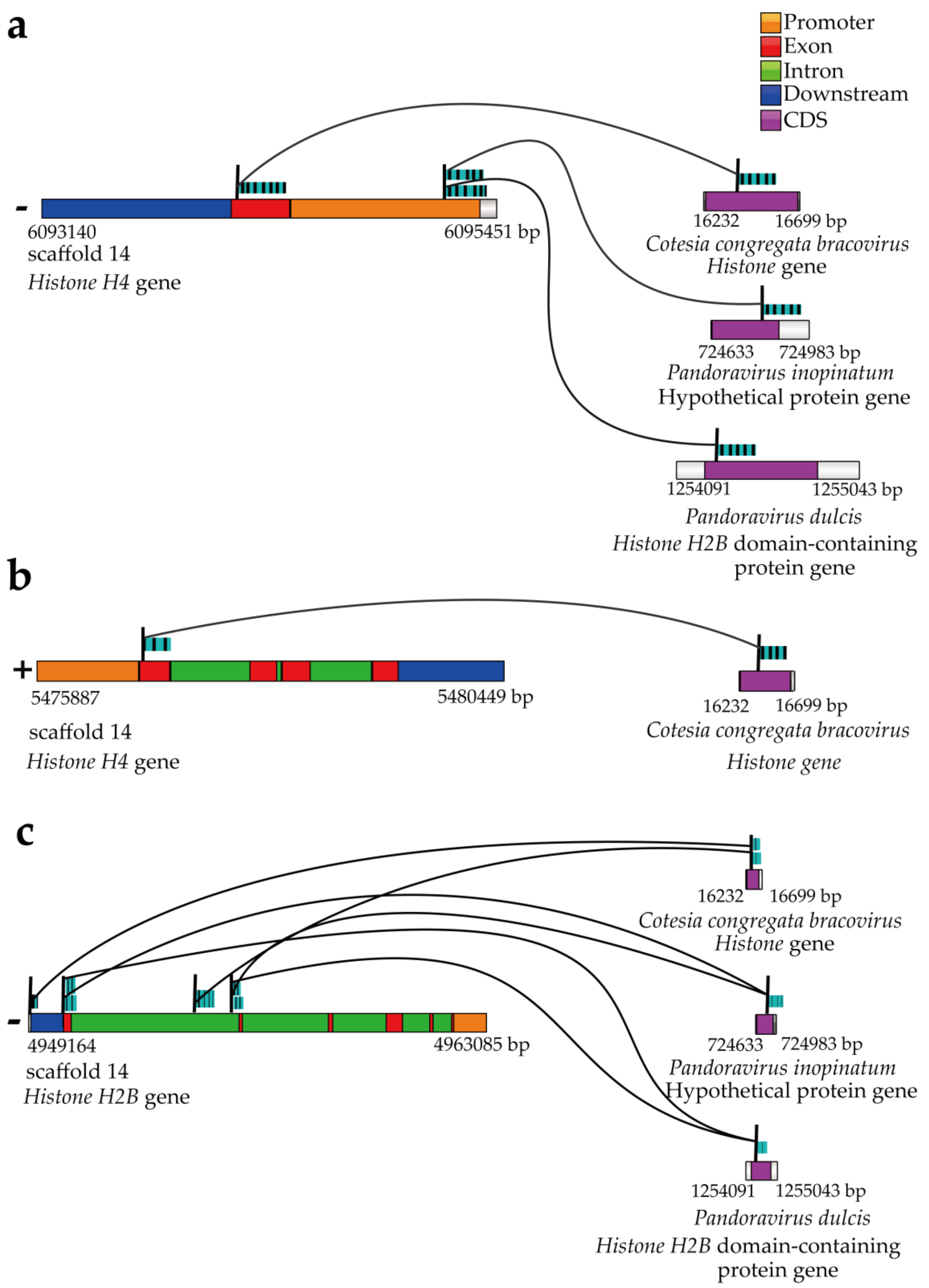

Figure S4. The HFs of amphioxus genes containing the HFs with the highest confidence of long length and high alignment quality. The 5 HFs with the highest confidence were located in *Histone H4* and *Histone H2B* of the amphioxus and were homologous to *C. congregata bracovirus*. In addition to the HFs with the top longest length and the highest alignment quality, other HFs within the gene were also shown. There were multiple viral DNA fragments homologous to one DNA fragment of amphioxus and one viral DNA fragment were possibly homologous to multiple DNA fragments of amphioxus. The bars on the left represent the amphioxus genome; different colors represent different genomic regions of

amphioxus; the dark green bars with vertical line represent the HFs of amphioxus. The bars on the right represent viral genomes; different colors represent different genomic regions of virus; the dark green bars with vertical line represent viral HFs. Yellow represents promoter regions in the amphioxus genome; red represents exon regions in the amphioxus genome; green represents intron regions in the amphioxus genome; blue represents downstream regions in the amphioxus genome; purple represents CDS regions in viral genomes. (a) The HFs of *Histone H4* on scaffolds 14. (b) The HFs of *Histone H4* on scaffolds 14. (c) The HFs of *Histone H2B* on scaffolds 14.
